# Brain morphology in adolescent girls with first-onset anorexia nervosa

**DOI:** 10.1007/s00787-025-02715-8

**Published:** 2025-04-23

**Authors:** Janneke S. P. Kovoor, Katrien F.M. Bracké, Tonya White, Gwen C. Dieleman

**Affiliations:** 1 Department of Child and Adolescent Psychiatry/Psychology, Erasmus MC-Sophia Children’s Hospital, Rotterdam, The Netherlands; 2 Department of Radiology and Nuclear Medicine, Erasmus MC, Rotterdam, The Netherlands; 3 Section of Social and Cognitive Developmental Neuroscience, National Institute of Mental Health, 10 Center Drive, CRC/4-2352, MSC 1348, Bethesda, MD 20892-1276 USA

**Keywords:** Anorexia nervosa, Adolescents, T_1_-weighted MRI, Brain volume, Body mass index, Eating disorder symptoms

## Abstract

This study aimed to assess whether a young, first-onset cohort of girls with anorexia nervosa (AN) demonstrate similar findings of reduced gray and white matter volume reductions as reported in older women with chronic AN. We compared adolescents and young adults with first-onset AN to typically developing (TD) girls and investigated associations between brain volume measures and clinical symptoms. Whole-brain T_1_-weighted structural imaging was performed in 58 young persons with AN and 63 age, sex-, and education-matched TD participants. Participants with AN were further divided into underweight (undAN) and restored weight (restAN) groups based on body mass index length/weight standard deviation score (BMI-SDS). Eating disorder symptoms were measured by the Eating Disorder Examination (EDE) and the Eating Disorder Inventory-3 (EDI-3). Differences in brain volume measures were analyzed using separate linear regression analyses. Within the AN group, associations between brain volumes, BMI-SDS, and EDE/EDI-3 scores were analyzed. The undAN group showed significant reductions in total and cortical gray matter volumes compared to the TD group, primarily in the frontal and parietal cortices. No significant associations were found between brain volume and BMI-SDS or EDE/EDI-3 scores. Frontal and parietal cortices in adolescents and young adults with undAN are particularly affected by malnutrition, showing gray matter volume reductions even in early stages of AN. Longitudinal studies are needed to explore the effects of malnutrition on brain development and its association with clinical features of AN over time.

**Supplementary Information:**

The online version contains supplementary material available at 10.1007/s00787-025-02715-8.

## Introduction

Anorexia nervosa (AN) is a severe psychiatric disorder with a typical onset in adolescence [1]. It is characterized by restricted food intake, a fear of weight gain, and a distorted perception of body image [2]. In clinical practice, treatment primarily focuses on weight restoration and reduction of eating disorder symptoms [3]. However, there is less awareness of the potential neurobiological consequences associated with AN-related malnutrition. Previous studies have shown considerable differences in structural brain morphology during the acute phase of AN [4], highlighting the need for greater awareness in clinical practice of potential brain alterations in individuals with AN. This is particularly important given that AN typically emerges during adolescence, a critical period for brain development [5, 6].

Structural brain changes observed in acutely ill AN patients include reduced global and local gray matter volumes, reduced white matter volumes, and increased cerebrospinal fluid (CSF) volume [7, 8]. These findings are present in both adolescent and adult patient populations, although these brain volume measures appear to be more dramatically reduced in adolescents with AN [9, 10]. These brain volume reductions occur irrespective of the hydration status of the patients [11, 12]. The mechanisms underlying structural brain changes in AN remain poorly understood. In addition, previous work exploring the association between age and brain morphology in AN reported lower gray matter volume with increasing age, in comparison with typical neurodevelopment [13].Volume reductions are frequently reported in both cortical and subcortical gray matter, and brain regions that show reductions in brain volume are often involved in functions that are known to be disordered in AN.

Several studies have investigated the relationship between brain volume alterations and clinical features of AN. Martin et al. observed reduced gray matter volumes in the medial and posterior cingulate cortex, structures which are involved in body- and visuospatial orientation and suggested that these reductions could be related to AN patients’ distorted perception of their own bodies [14]. A subsequent meta-analysis of structural and functional MRI in AN by Su et al. similarly identified volume reductions within the cingulate cortex, and additionally within the inferior parietal lobe and precuneus, structures which have been linked to abnormal reward processing related to food intake in AN [15, 16].

Other studies have investigated the relationship between brain volume and the main clinical features of AN.Walton et al. found a positive association between BMI and subcortical volumes in both underweight and weight-restored adolescents and adults with AN [7]. Longitudinal studies have shown that brain volume reductions in AN are largely reversible with weight restoration [11, 14, 17]. These findings indicate that AN patients in study populations should be either stratified based on the stage of their illness, or that BMI should be controlled for when analyzing structural neuroimaging data. The study by Walton et al. combined data from multiple different cohorts to create the largest cross-sectional structural neuroimaging AN study to date [7]. However, since adult and adolescent data were aggregated, no conclusions could be drawn that were specific to adolescents. The relationship between brain volume alterations and eating disorder symptoms has been studied less frequently. One study found a positive association between eating disorder symptoms and regional gray matter volumes in adult underweight AN participants [18], while another study also investigating these variables found no association in a similar population [19]. Another study conducted in adolescents newly diagnosed with an eating disorder found that brain volumes were both positively and negatively correlated with eating disorder cognitions [20].These findings indicate that the connection between brain volume and clinical symptoms in AN remains poorly understood and highlights the need for further research.

Despite previous studies on brain morphology in individuals with AN, several knowledge gaps remain. First, most studies have focused on individuals with AN who were more severely ill and had a longer illness duration [7, 9, 14, 21], and there is less knowledge regarding potential brain alterations during the early stages of AN in adolescent girls. As AN typically occurs in adolescence, it is important to improve our understanding of the effects of AN on the developing brain in the early stages of the disease. Second, as far as we know, there are no studies that assess the relationship between brain volume alterations and eating disorder symptoms in adolescents with first-onset AN, nor between brain volume alterations and BMI. Given that eating disorder symptoms and BMI are the main clinical outcomes of AN, it is important to improve our knowledge on this association, with the potential to ultimately guide clinical practice. Furthermore, limited research has focused on adolescents experiencing their first episode of AN, who may not yet show the long-term effects of chronic starvation of the brain. As AN typically develops during adolescence, a critical period of brain development, more research is needed to investigate the effects of starvation on the developing brain.

Within the backdrop of the current literature on AN, the primary aim of our study is to investigate differences in brain morphology in a large clinical case-control sample of female adolescents and young adults with first-onset AN, aged 12–22 years, and age-, sex-, and education-matched typically developing (TD) individuals. The AN participants were divided into subgroups of underweight (undAN) and weight-restored (restAN) patients to examine the effect of weight restoration on brain structure. We hypothesized that global brain volume measures would be reduced in the undAN group compared to both restAN and TD participants. Further, we hypothesized that brain volumes in the restAN group would not be reduced compared to TD. Given that our study focuses exclusively on young persons, we were able to study the effects of malnutrition on brain structure in a population that is currently undergoing brain development [5, 6, 22], which may differ from alterations observed in adults, providing further insight into the etiology of the disorder. Second, we aimed to investigate the association between brain volume and BMI-SDS. Based on previous research, we expected to find a positive association between BMI-SDS and brain volume measures in AN participants. Third, we aimed to test whether there was an association between brain volume measures and eating disorder symptoms. Due to relatively inconsistent findings in previous studies, we do not have specific hypotheses related to this aim.

## Methods

### Design and study population

**Fig. 1:**
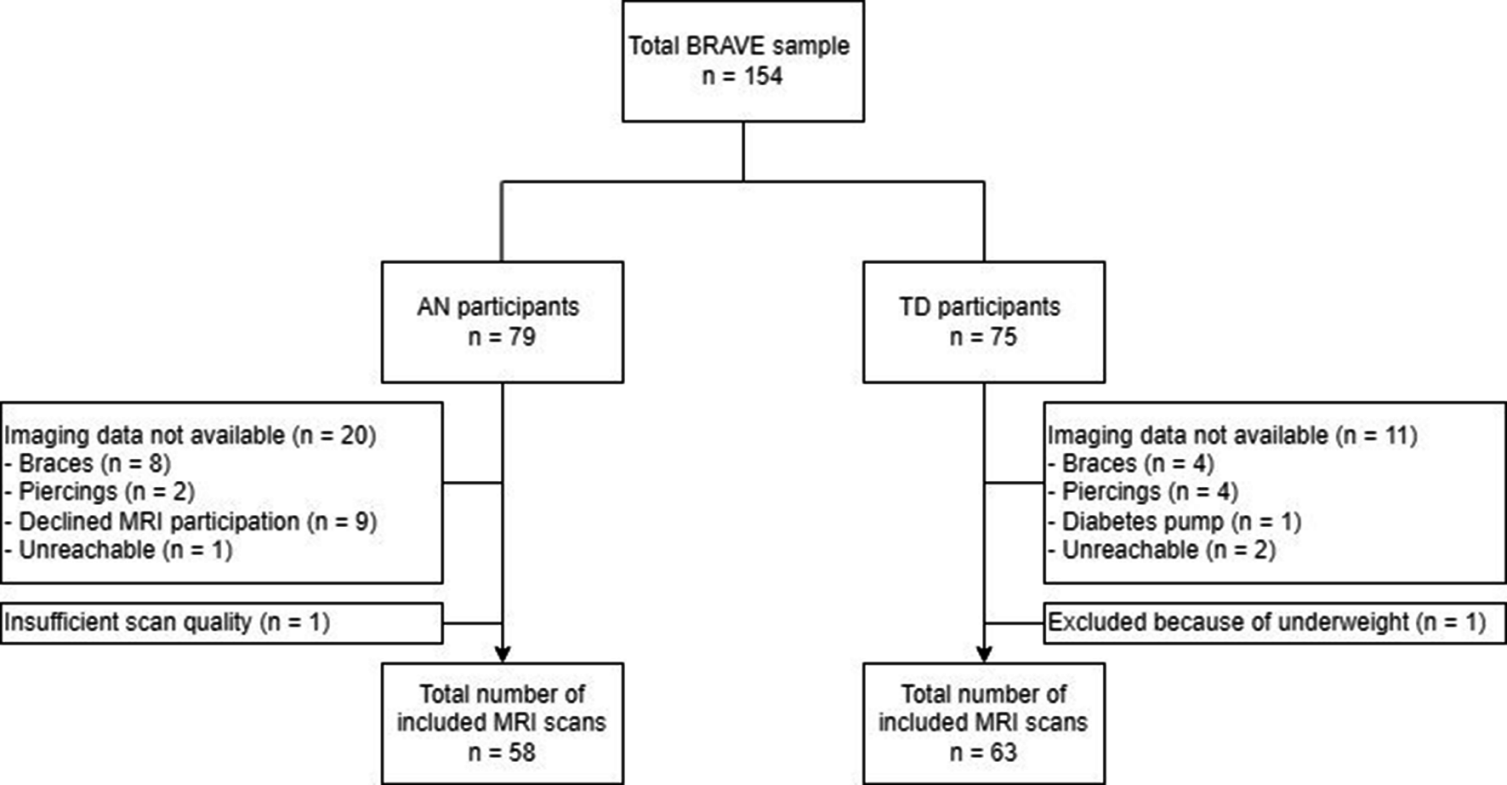
Flowchart of inclusion of study population. BRAVE = Brain function and attentional processes in adolescent anorexia nervosa; AN = anorexia nervosa; TD = typically developing

The study was embedded in the BRAVE study, a first-onset AN longitudinal case-control cohort study with a repeated measures design conducted within the Erasmus Medical Center - Sophia Children’s hospital (Erasmus MC-Sophia) in Rotterdam. Detailed information about the recruitment procedure and study design of the BRAVE study is published elsewhere [23]. In brief, individuals with first-onset AN, defined as their first lifetime episode of AN, diagnosed within 12 months of the date of inclusion, were recruited from 16 mental health institutions in the Netherlands through their health care providers, advertisements on social media and through patient organizations. TD participants were recruited through AN participants or social media advertisements. In total 154 young persons were included in the study: 79 participants with AN and 75 TD participants. In this article we define young persons as female adolescents aged 12 to 19 and female young adults aged 20 to 22. AN participants were eligible for inclusion if they were females aged 12–22 years, diagnosed with AN according to the DSM-5 criteria within the past year. TD participants were matched on sex assigned at birth, age, and educational level and were required to have a healthy weight (BMI-SDS between > -1.3 and < + 1.3). Exclusion criteria were the presence of a psychotic-, neurologic-, or a substance abuse disorder, severe motor or sensory disturbances, IQ < 70 measured by an intelligence test, and insufficient Dutch language skills. The majority of the participants underwent an MRI scan (58 AN participants and 64 TD participants). A flowchart of the study sample is presented in Fig. 1. Exclusion criteria for MRI scanning include general contra-indications for MRI scanning (e.g. braces, piercings), and severe head injury with loss of consciousness. One MRI scan of a TD participant was excluded due to not meeting the healthy weight criteria, resulting in a final sample of 121 participants (AN = 58; TD = 63). The MRI scan and clinical measures were assessed during two visits, conducted in random order at the Erasmus MC-Sophia. Written informed consent/assent was obtained from all participants and their parents for those under the age of 16. The BRAVE study was approved by the Medical Ethical Committee of the Erasmus MC-Sophia (MEC 2016 − 194/NL55175.078.16).

### Measures

#### Clinical measures

*BMI-SDS* is a BMI measure that has been adjusted for age and sex. It was calculated using an online growth calculator [24] and was based on participant height and weight measurements that were recorded on the day of MRI scanning. Dutch growth curves based on the WHO thinness grades were used to adjust BMI for age [25]. A BMI SDS of -1, for example, indicates that a participant’s BMI is one standard deviation below the average BMI for her age and sex in a reference population. As an example, the mean BMI-SDS for the undAN group is -2.26, which would be equivalent to an adult being at approximately 70% of their ideal body weight.

*Eating disorder symptomatology* was evaluated using the Eating Disorder Examination-12th edition (EDE) and the Eating Disorder Inventory-3 (EDI-3). The EDE is an interview consisting of 35 questions evaluating participant’s concerns related to food, body shape and weight. It is considered the gold standard in clinical practice, with high internal consistency reported for both total and subscale scores (α = 0.92) [26, 27]. At the time of baseline data collection, EDE interviews were conducted with participants by a trained researcher. The EDI-3 is a self-report measure assessing psychological and behavioral symptoms of eating disorders, consisting of 91 questions organized into 12 subscales. Satisfactory internal consistency has been reported for EDI-3 subscale scores (α > 0.80) [28].

*Depressive symptoms* were assessed using the Beck Depression Inventory-Second Edition (BDI-II), a self-report questionnaire that measures the severity of depressive symptoms over the past week [29]. The BDI-II includes 21 items rated on a 4-point likert scale, with higher total scores indicating more severe depressive symptoms. The internal consistency of the BDI-II is high (α = 0.9) [30].

*Anxiety symptoms* were evaluated with the Screening for Child Anxiety Related Emotional Disorders (SCARED) questionnaire, which is based on DSM-IV-TR criteria [31]. The self-report questionnaire consists of 69 items rated on a 3-point likert scale, with higher scores indicating more anxiety symptoms. The internal consistency of the SCARED questionnaire ranges from moderate to high (α = 0.74–0.93) [32].

#### MRI acquisition

MRI imaging was conducted using a GE 3 Tesla Scanner with an 8-channel head coil, and sequences include T_1_ weighted structural images with a resolution of 1-mm^3^. All participants included in this study were scanned using the same system and sequences. The image sequence parameters are as follows: TR = 10.3 ms, TE = 4.2 ms, TI = 350 ms, NEX = 1, flip angle = 16°, readout bandwidth = 20.8 kHz and matrix size of 256 × 256. Scan quality was assessed by trained researchers using a systematic rating scale [23, 33]. Morphological analysis of MRI scans was done using FreeSurfer. Parcellation of the cortex was based on the Desikan-Killiany atlas [34]. The global brain volume measures are defined as follows: total brain volume, total gray matter volume, total white matter volume, and CSF volume. Total white matter volume was calculated as the sum of cerebral white matter volume and cerebellar white matter volume. If any significant differences were found in global brain volume measures, we further analyzed which local brain regions were driving these results.

### Data analysis

First, the distribution of variables was evaluated. Measures of EDE, BDI-II, and the SCARED were log-transformed to approach a normal distribution. Second, we performed linear regression analyses to test for differences in the global brain volume measures between AN and TD participants. The linear regression analyses were performed with group (AN/TD) as the independent variable and global brain volume measures as dependent variables in separate models. Given that a subset of the AN participants were already weight-restored at the time of MRI-scanning, we did an additional three-group comparison: underweight AN (undAN) with BMI-SDS < -1.3, weight-restored AN (restAN) with BMI-SDS > -1.3, and TD participants. We used a stepwise whole-brain approach to our analysis and analyzed differences in brain volume between all possible pairs of groups. All analyses were adjusted for age. A sensitivity analysis was done by adding BMI-SDS as an additional covariate to the linear regression models when testing for differences in the global brain volume measures between undAN, restAN and TD participants. Follow-up analyses were performed using the same set of independent variables, but with the following brain volumes as dependent variables: cortical gray matter volume, cerebral white matter volume, subcortical gray matter volume, and total cerebellar volume. A secondary three-group analysis was conducted for all cortical brain volumes, as defined by the Desikan-Killiany Atlas. Intracranial volume was included as an additional covariate in these models. We have conducted a sensitivity analysis with menstrual status as a covariate. We defined menstrual status as having had a period within the last three months. Third, to investigate the association between BMI-SDS and brain volume, and age and brain volume, we performed linear regression analyses with BMI-SDS and age as independent variables and the global brain volume measures as dependent variables. This analysis was conducted separately within the AN group overall, as well as in each of the AN subgroups and the TD group. Fourth, to investigate the association between eating disorder symptoms and brain volume, we performed linear regression analyses within the AN group. Separate models were created using either EDE or EDI-3 total score as the dependent variable, with each of the global brain volume measures as independent variables and age as a covariate. A second analysis was conducted that included BMI-SDS as an additional covariate. Given the known negative correlation between eating disorder symptoms and BMI-SDS, we aimed to explore the relationship between eating disorder symptoms and brain volume measures independently of the effect of BMI-SDS. Statistical analyses were conducted in R version 4.2.2. A significance threshold of *p* < 0.05 was maintained. Correction for multiple testing was done using the False Discovery Rate (FDR) method [35]. We applied FDR correction accounting for all analyses performed, which was in total 8 comparisons per pair. Based on the results of this analyses, we analyzed the 34 regions of the cortex, resulting in 34 comparisons being corrected using FDR.

## Results

### Participant characteristics

Participant characteristics are shown in Table 1. In total 121 participants were included in the analyses. The majority of AN participants had the restrictive subtype (81.30% in the undAN group and 76.90% in the restAN group). The TD, undAN and restAN groups are similar based on age, maternal education level, and ethnicity. As expected, BMI-SDS was reduced in the undAN group compared to both restAN and TD groups, and reduced in the restAN compared to the TD group. The undAN and restAN groups scored higher on questionnaires evaluating eating disorder symptoms compared to TD. The undAN and restAN groups had a comparable illness duration and use of psychotropic medications. Furthermore, the undAN and restAN groups had more depression, anxiety, and obsessive-compulsive symptoms compared to the TD group.

**Table 1:** Participant characteristics

	n	undAN^a^ (*n* = 32)		restAN^b^ (*n* = 26)		TD^c^ (*n* = 63)		*p*
		Mean (SD); n/% (n)	Range	Mean (SD); n/% (n)	Range	Mean (SD); n/% (n)	Range	
**Demographic Characteristics**								
Age (years)	121	17.03 (2.27)	12.64–21.60	16.97 (1.94)	15.03–21.75	17.56 (2.10)	13.37–22.27	0.358
Maternal education level, n (%)^d^	110							0.425
Low		0 (0.00)		0 (0.00)		0 (0.00)		
Middle		15 (48.39)		14 (60.87)		25 (44.64)		
High		16 (51.61)		9 (39.13)		31 (55.36)		
Ethnicity, n (%)^e^	119							0.781
Dutch		28 (87.50)		23 (95.83)		53 ( 84.13)		
(other) Western		3 (9.38)		1 (4.17)		6 (9.52)		
Non-Western		1 (3.13)		0 (0.00)		4 (6.35)		
**Clinical Characteristics**								
AN subtype, n (%)								0.688
Restrictive subtype Binge-purge subtype		26 (81.30) 6 (18.8)		20 (76.90) 6 (23.10)		-		0.688
BMI-SDS^f^	121	-2.26 (0.72)	-4.11– -1.33	-0.26 (0.95)	-1.28–2.35	0.51 (0.91)	-1.20–3.33	< 0.001^xy^
Duration of illness (days)	115	181.71 (133.16)	25.00-436.00	209.46 (122.35)	19.00-486.00	-		0.441
EDE total score^g^	121	3.55 (1.05)	1.11–5.35	3.75 (1.17)	0.96–5.32	0.35 (0.43)	0.00-1.79	< 0.001^x^
EDI total score^h^	115	159.81 (41.26)	77.00–238.00	152.90 (40.28)	1.00-259.00	45.07 (28.47)	3.00-160.00	< 0.001^x^
Psychotropic medication use, n (%)	121	4 (12.50)		5 (19.23)		5 (7.94)		0.289
Comorbidities								
BDI-II total score^i^	108	30.62 (11.84)	12.00–56.00	25.89 (11.52)	8.00–53.00	4.80 (5.43)	0.00–28.00	< 0.001^x^
SCARED total score^j^	108	42.79 (21.61)	9.00–88.00	44.21 (19.70)	15.00–85.00	22.37 (14.90)	3.00–71.00	< 0.001^x^
CY-BOCS total score^k^	82	13.05 (13.15)	0.00–33.00	7.05 (8.18)	0.00–25.00	1.62 (4.61)	0.00–21.00	< 0.001^x^
Y-BOCS total score^l^	36	10.88 (11.19)	0.00–30.00	11.86 (11.98)	0.00–26.00	1.52 (4.32)	0.00–18.00	0.009^x^

^a^ undAN = underweight anorexia group

^b^ restAN = restored weight anorexia group

^c^ TD = typically developing group

^d^ Low: primary education and lower secondary education; Medium: higher general secondary education; High: higher vocational secondary education and higher academic education

^e^ Ethnicity is based on country of birth of mother

^f^ BMI-SDS = body mass index length/weight standard deviation score

^g^ EDE = Eating Disorder Examination-12th edition

^h^ EDI = Eating Disorder Inventory-3

^i^ BDI-II = Beck’s Depression Inventory-2

^j^ SCARED = Screen for Child Anxiety Related Disorders

^kl^ (C)Y-BOCS = (Children’s) Yale-Brown Obsessive-Compulsive Scale

^x^ p-value < 0.05 post hoc comparison undAN and restAN different from TD participants

^y^ p-value < 0.05 post hoc comparison different between undAN and restAN

### Brain volume differences

**Fig. 2:**
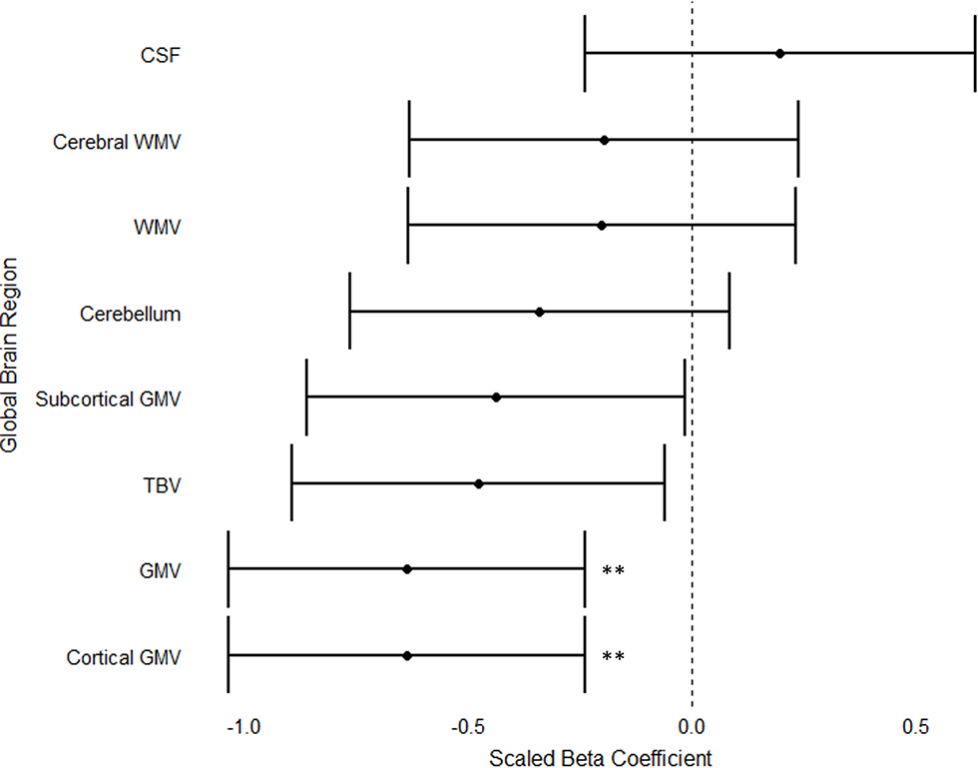
Differences in global brain volumes in undAN vs. TD participants. WMV = white matter volume; GMV = gray matter volume; TBV = total brain volume; CSF = cerebrospinal fluid volume. Volumes that reached significance after FDR-correction are indicated with asterisks (*) and significance levels of p-values are defined as follows: * *p* < 0 0.05; ** *p* < 0.01; *** *p* < 0.001

The two-group analysis evaluating differences in global brain volume between the AN and TD groups revealed a reduction in total gray matter volume in the AN group compared to TD group (Table S1, Online Resource). The three-group analysis demonstrated that total gray matter volume and cortical gray matter volume were reduced in the undAN group relative to the TD group after FDR-correction (Fig. 2). No difference in any of the global brain volume measures was observed between the restAN and TD groups or between the undAN and restAN groups.

The sensitivity analysis including BMI-SDS as an additional covariate revealed that total gray matter volume and total brain volume were reduced in the undAN group compared to TD participants (*p* = 0.009 and *p* = 0.015, respectively) (Table 4, Online Resource). We performed a sensitivity analysis with menstrual status as an additional covariate. We found that gray matter and total brain volume were reduced in the undAN group compared to both the TD group and the restAN group. These results are conform the findings of the original analysis (Table S7, Online Resource).

**Fig. 3:**
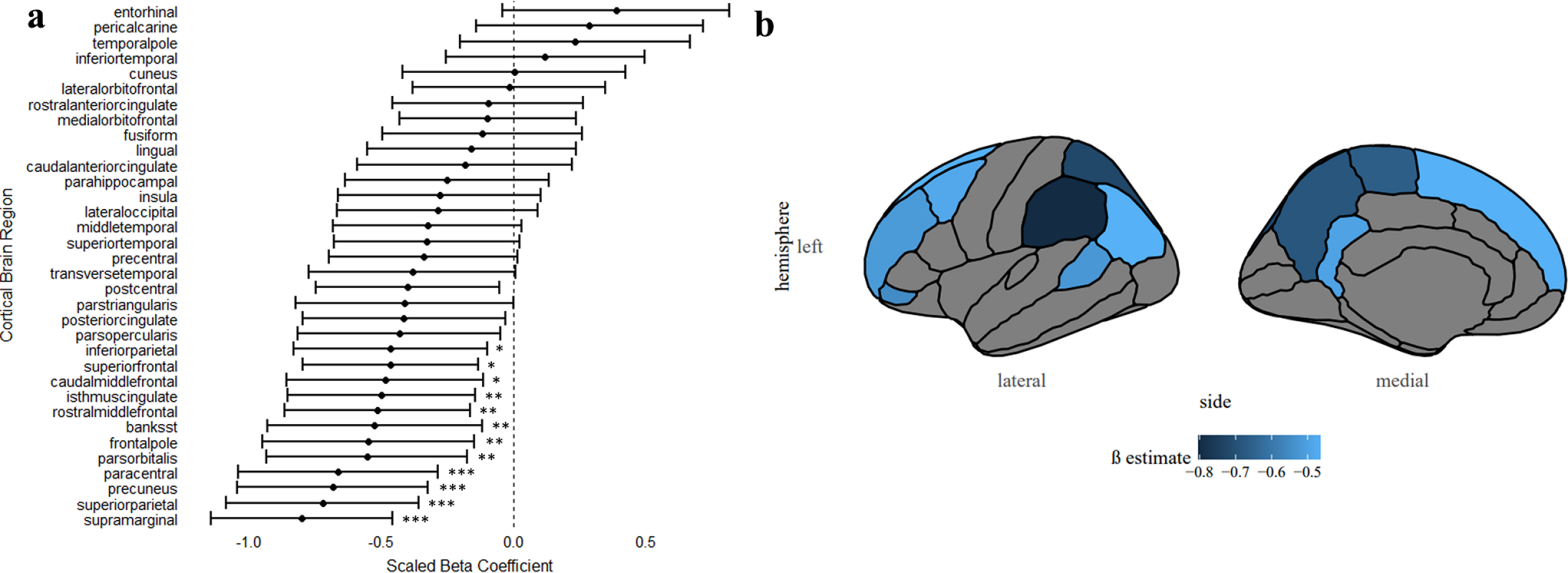
**a** Differences in cortical brain volumes in undAN vs. TD participants. Cortical brain regions as defined by Desikan et al. Volumes that reached significance after FDR-correction are indicated with asterisks (*) and significance levels of p-values are defined as follows: * *p* < 0 0.05; ** *p* < 0.01; *** *p* < 0.001. **b** Reductions in cortical brain volumes between underweight AN participants and TD participants are highlighted in blue. The β estimates, averaged across the left and right hemispheres, are depicted on the left hemisphere

A secondary 3-group analysis (Table S2, Online Resource) was conducted to identify volume alterations across the 34 regions of the cortex. Volume reductions were observed in several cortical regions in the undAN group compared to TD group (Fig. 3a). Regions that remained significant after FDR correction are located primarily within the frontal and parietal lobes and are presented in Fig. 3b Four regions (frontal pole, β = -342.24, *p* = 0.001; precuneus, β = -1771.95, *p* = 0.002; rostralmiddlefrontal, β = -2481.77, *p* = 0.006; supramarginal, β = -2662.65, *p* = 0.001) were reduced in volume in the undAN group compared to restAN after FDR-correction (Table S3, Online Resource).

### Association between brain volume reduction and BMI-SDS

We found a negative association between BMI-SDS and CSF volume in the AN group overall (β = -39.34, *p* = 0.03) and in the restAN group (β = -85.64, *p* = 0.02). This suggests that higher BMI-SDS is associated with lower CSF volume. No further associations between BMI-SDS and the global brain volumes were found in the AN group or in the undAN and restAN subgroups (Table S5, Online Resource).

### Association between brain volume reduction and age

We found a negative association between age and gray matter volume in the AN group overall and in the undAN and TD subgroups, indicating that gray matter volume decreases with increasing age (Table S5, Online Resource). There was no significant association between age and gray matter volume in the restAN subgroup. A scatter plot was created with these results, allowing for a visual comparison of the slopes and intercepts across the subgroups (Table S8, Online Resource). There was an association found between age and gray matter volume, and age and total brain volume in the AN group and undAN group. We observed that with increasing age, brain volumes decrease. There was no association between age and brain volume in the restAN group.

### Association between brain volume reduction and eating disorder symptoms

Neither the univariate model nor the multivariate model that included BMI-SDS as a covariate revealed any statistically significant associations between brain volume and eating disorder symptoms within the AN group (Table S6, Online Resource).

## Discussion

This study examined brain volume measures in female adolescents and young adults with first-onset AN, compared to age-, sex-, and education-matched controls. Furthermore, we investigated whether there was an association between brain volume measures and two key clinical outcomes of AN, namely BMI and eating disorder symptoms. We found that total gray matter volume was reduced in undAN participants compared to TD participants, which is in line with previous findings [7, 36–38]. Additionally, a secondary analysis of the cortex revealed that gray matter volume reductions existed primarily within the frontal and parietal lobes, including the superior and inferior parietal gyri and prefrontal cortex. There were no differences in brain volume measures between undAN and restAN participants. Contrary to our initial hypothesis, we did not find an association between brain volume measures and clinical outcomes, i.e. BMI and eating disorder symptoms.

Our observation that volume reductions are localized in the frontal and parietal cortices in undAN is interesting because these are precisely the regions of the cortex that, in the trajectory of typical development, are in the process of maturation during adolescence and early adulthood [5, 6, 22]. Previous studies have similarly identified volume reductions in these regions in acutely ill AN populations [14, 21, 36, 39]. Walton et al. found that “densely connected hub regions’’ of the brain, i.e., areas that form networks with other brain regions, were particularly reduced in volume in undAN patients [7]. The superior and inferior parietal gyri are two such regions, responsible for functions such as the processing of proprioceptive information, which are often disordered in AN [40, 41]. The prefrontal cortex is another such “hub” region, involved in executive functions, including decision-making and problem-solving skills. These have also been shown to be disordered in AN [9, 14]. It is well established that increased myelination and synaptic pruning are essential to brain development and that these processes are particularly active in the frontal and parietal lobes during adolescent neurodevelopment, including in the prefrontal and posterior parietal cortices [5, 6, 22]. Previous research has shown that brain regions exhibiting high levels of metabolic activity correspond to areas that are undergoing the most rapid maturation [42, 43]. These findings are further supported by a study by Bahnsen et al., in which large differences in cortical thickness were observed between AN and TD groups in regions with high expression of cells involved in the typical process of brain maturation, including areas within the frontal and parietal cortices [44]. Furthermore, it has been observed that gray matter volume reductions are greater in acutely-ill adolescent AN patients compared to acutely-ill adult patients [10, 45]. These findings, taken together with the results of the present study, indicate that the developing frontal and parietal cortices are particularly susceptible to the effects of malnourishment in adolescents. We did not find differences in brain volume measures between undAN and restAN participants. According to the literature, brain volume reductions are associated with BMI-SDS, with the most pronounced reductions observed in severely underweight patients [7]. Therefore, it is possible that the reductions in brain volume in the restAN participants have already been reversed. The rapid restoration of brain volume in restAN participants may be attributed to the increased brain plasticity during adolescence [46], highlighting the importance of timely intervention in AN. This suggests that partial weight recovery might be sufficient to normalize brain volume in young individuals with AN.

Though we initially observed reduced subcortical volume in the undAN group compared to TD, this result did not survive FDR-correction. In addition, we did not observe differences in cerebral white matter volume and cerebellar volume in the AN groups compared to the TD group. Furthermore, we did not observe significantly greater CSF volumes in our AN patients compared to the TD participants, which contrasts with previous studies [12, 38, 45, 47]. Studies that report subcortical [7, 14, 21, 36, 39] and white matter [12] volumetric differences in AN compared to TD participants were often conducted in hospitalized patients, who were more severely ill and with longer average illness durations than the participants in our study. It is possible that volume differences that have previously been identified were not found in the current analysis because our study consists of girls with first-onset AN with a short-lived illness. It is possible that alterations in some regional brain volume measures, such as global white matter volume, subcortical gray matter volume, and CSF require a longer duration of malnutrition to manifest. In addition, our AN sample was divided into two subgroups, resulting in a moderate sample size. Therefore, our study may have insufficient power to detect significant differences in subcortical, white matter, and CSF volumes.

Furthermore, our sensitivity analysis including menstrual status as an additional covariate did not deviate from the original analysis, indicating that menstrual status does not affect brain volume in our AN population.

Previous studies have demonstrated an association between BMI and brain volume in individuals with AN [7, 14, 45, 48], specifically in relation to cortical and subcortical volumes. We did not find an association between BMI-SDS and either total brain volume, gray matter volume, or white matter volume in our AN population. This may be due to our population being less severely ill relative to those of previous studies [7, 14, 21, 36, 39]. Another possible explanation for this finding is that a large proportion of our sample was weight-restored, which may have reduced the statistical power to detect an association between brain volume and BMI-SDS. However, we did find a negative relationship between CSF volume and BMI-SDS in both the total AN group and the restAN group, indicating that higher BMI is associated with lower CSF volume. This finding is in line with previous studies [38].

We conducted an additional analysis to explore the relationship between age and brain volume. In both the undAN and TD groups, we observed a decrease in gray matter volume with increasing age, which is consistent with the typical trajectory of brain development in adolescents [48]. However, gray matter reduction was greater in the undAN group compared to the TD group, suggesting that the typical age-related reduction in gray matter is exacerbated by starvation effects during the acute phase of AN. Additionally, the rate of decline in gray matter volume with age in the restAN group, though not statistically significant, was less than that in both the undAN and TD groups. This non-significant result may be due to greater variability in the data from the restAN group. Nevertheless, it remains noteworthy that gray matter volume in the restAN group was trending toward levels observed in the TD group, suggesting that brain volume reduction seen in early-onset AN may be reversible.

Assessing the relationship between eating disorder symptoms and brain measures in girls with AN, we did not find associations between global brain volumes and total scores of either the EDE or EDI-3. This result is not necessarily surprising, however, since findings in the literature are inconsistent with respect to this question in studies that have analyzed adult populations rather than adolescents [18, 19]. As far as we know, no studies have investigated associations between EDI-3 scores and brain volumes.

### Strengths and limitations

The strengths of our study include the unique sample of adolescent girls with a first episode of AN who received the diagnosis less than a year prior to inclusion. Most previous neuroimaging studies in AN have included patients with a longer illness duration, without necessarily focusing on those having their first episode of AN. In the largest structural neuroimaging study done to date in AN patients, the average illness duration was 5 years [7]. A second strength of our study is our sample allowed us to compare well matched samples of AN and TD participants and to further subdivide the AN patients into underweight and weight-restored groups.

Several limitations need to be taken in consideration. First, this is an observational cross-sectional study. Thus, these results do not suggest direct causation, but are limited to indicating the relationships between variables. Between time of diagnosis and baseline data collection, the clinical measures of a portion of the AN participants improved, likely as the result of undergoing treatment. BMI is one such clinical measure and is why it was necessary to differentiate between undAN and restAN participants. It is therefore possible that our data set did not have enough power to detect volume alterations in other areas of the brain in AN. It is also possible that existing changes in brain volume had become less severe in the time between diagnosis and baseline data collection. However, if this is the case, it would also suggest that the areas that we found to be reduced in volume are particularly sensitive to malnourishment. Second, our approach using parcel-based volumes may be less suitable for detecting smaller regions potentially affected in AN. However, an advantage of this method is that averaging brain volumes across larger areas, especially when considering the possibility of more global differences typically seen in AN, generally reduces noise compared to vertex-wise techniques. Third, as we did not have information on sex steroids, we could not completely rule out hormone-related structural changes in the brain. However, the results of our sensitivity analysis indicate that menstrual status does not have an effect on brain volume in our AN population, therefore we can reasonably conclude that the structural changes in the brain are not purely hormone-related. Additionally, due to the multiple tests in the BRAVE study, participants had to be both mentally and physically able to complete data collection, which could have resulted in the exclusion of individuals who were more severely ill.

## Conclusion

In conclusion, our findings provide evidence for gray matter volume reductions, primarily localized to the frontal and parietal lobes in underweight girls with AN compared to weight-restored AN and TD girls. Frontal and parietal cortices are actively under development in the adolescent brain and thus have higher energy demands, and seem particularly susceptible to malnourishment. This suggests that gray matter volume reductions develop quite rapidly with the onset of malnutrition. We did not observe brain volume differences in the restAN group compared to the TD group, suggesting that brain volume is unaltered in AN patients that are not underweight. Therefore, timely intervention in the course of AN may have the potential to prevent brain volume alterations at an important stage in brain development. Our findings reinforce the importance of early diagnosis and treatment of AN. Further research should be conducted to learn more about how malnutrition experienced by AN patients is affecting the metabolic processes of maturing brain regions and neural circuitries. Longitudinal studies with both structural and functional neuroimaging measures would allow for further insight into AN patients’ response to treatment and recovery process, and factors that are potentially correlated with disease course.

## Electronic supplementary material

Below is the link to the electronic supplementary material.

Supplementary Material 1

## Data Availability

No datasets were generated or analysed during the current study.
